# NOD2 is highly expressed in Behçet disease with pulmonary manifestations

**DOI:** 10.1186/1476-9255-9-3

**Published:** 2012-02-13

**Authors:** Kamel Hamzaoui, Hanadi Abid, Anissa Berraies, Jamel Ammar, Agnès Hamzaoui

**Affiliations:** 1 Division of Histology and Immunology, Department of Basic Sciences, Medicine School of Tunis, Tunis El Manar University, 15 Rue Djebel Lakdar, 1007 Tunis, Tunisia; 2 Division of Pulmonology, Department of respiratory diseases and the Unit Research "Homeostasis and Cell dysfunction (99/08-40), Tunis El Manar University and A. Mami Hospital, Ariana, Tunisia

**Keywords:** NOD2, TLRs, T-bet, Behçet Disease, Inflammation

## Abstract

**Background:**

Excessive Th1 cells and TLRs functions are involved in the pathogenesis of Behcet's disease (BD) in response to bacterial antigens. NOD2, an intracellular pathogen recognition sensor, modulates innate defence to muropeptides derived from various bacterial species. To further define a role for NOD2 in BD, we analysed NOD2 transcriptional responses in BAL-MNC from BD patients with pulmonary manifestations.

**Methods:**

We analysed NOD1, NOD2, T-bet and TLRs mRNA expression with real-time polymerase chain-reaction in BAL cells obtained from 23 BD patients with pulmonary manifestations and their matched controls.

**Results:**

We found that NOD2 mRNA expression was highly up-regulated in BAL cells from BD and sarcoidosis patients compared to healthy control group (*P *= *0.001*). In BD patients, significant correlation was found between NOD2 and T-bet mRNA expression (r = 0.602; *P *= *0.0009*). In BAL from BD patients, NOD2 and T-bet mRNA expression were significantly correlated with BAL-lymphocytes (r = 0.485, *P *= *0.010*; r = 0684, *P *= *0.0001 respectively*). NOD2 in BD was also correlated with TLR 2(r = 0.444; *P *= *0.021*) and TLR 4 (r = 0.574; *P *= *0.001*) mRNA expression.

**Conclusion:**

Our results indicate that BAL-MNC from BD patients expressed NOD2 as a result of lung inflammation. TLRs and NOD2 synergize for the induction of proinflammatory cytokines. BAL inflammatory cells showed an increased Th1 situation as indicated by increased T-bet mRNA expression.

## Background

Behcet's disease (BD) is a systemic vasculitis with unknown aetiology. Immune dysregulation involving T and B cells with hyperreactive neutrophils, supposedly triggered by infectious agents, contribute to disease pathogenesis in addition to genetic predisposition [1-3]. Documentation of various atypical streptococcal species in oral flora of BD patients, clinical flares after dental procedures, and a good response to antibacterial treatment, have been considered as evidence for the role of Streptococcus in BD [4]. However, none of the microbial agents has been definitely proved to cause BD. Immunological disorders are important in BD pathogenesis [5]. T lymphocytes from patients with BD produced a particular inflammatory mediators pattern when stimulated with a bacterial superantigen [6-9]. Innate immunity was deeply investigated in BD patients [9,10]. Toll-like receptor (TLR)-expressing cells (TLR-2 and TLR-4) [9] and gamma delta T cells (TCRγδ) [11] have been involved in BD inflammatory reactions.

NOD-like receptors (NLRs) are a family of innate immune receptors that play key roles in host defence and inflammation. NLR genes have been preserved throughout evolution and at least 22 members are present in humans [12]. NOD2 is an intracellular receptor for the bacterial cell wall component muramyl dipeptide (MDP), and variants of NOD2 are associated with chronic inflammatory diseases of barrier organs (e.g., Crohn's disease, asthma, and atopic eczema) [12,13]. It is known that activation of NOD2 induces a variety of inflammatory and antibacterial factors. Truncated NOD2 proteins are encoded by mutations in the NOD2 gene that predispose individuals to inflammatory diseases [14].

To further define a role for NOD2 in BD with pulmonary manifestations, we analysed NOD2 mRNA transcriptional responses in BAL (broncho-alveolar lavage) and PBMC (peripheral blood mononuclear cells) harvested from BD patients with pulmonary manifestations, sarcoidosis patients (disease controls) and healthy controls. We correlated the transcriptional responses of TLR2 and TLR4 with NOD1 and NOD2 in BD patients.

## Methods

### Patients and cell isolation

The study group consisted of 27 BD patients (19 male, 8 female), 10 sarcoidosis patients and 23 healthy individuals. All of the BD patients (age 34 ± 10 years; range 17-56 years) fulfilled the international study group criteria for Behcet's disease [15], had disease duration for 1-9 years (mean ± SD: 5.8 ± 3.4). Twenty tree BD patients were never-smokers and 4 patients were ex-smokers as certified. All patients had active BD with pulmonary manifestations [16-18]. Clinical manifestations were eye lesions (14 patients: 51.85%) oral ulcer (27 patients: 100%) genital ulcer (18 patients: 66.67%) arthritis (16 patients: 59.25%) and vascular symptoms (12 patients: 44.45%). Pulmonary vascular abnormalities were as follows: asymptomatic functional abnormalities (8 patients), pulmonary artery aneurysm of varying signs (6 patients), pulmunary artery embolism (9 patients), and pulmonary venous abnormalities (4 patients). Remission was defined when clinical manifestations were lost (eye lesions, oral ulcer, genital ulcer and arthritis). Asymptomatic functional abnormalities diminished after corticosteroid treatment. BAL from ten patients with sarcoidosis acted as disease controls (7 men and 3 women; median age 37 years; range: 28-47). The diagnosis of sarcoidosis was determined in compliance with the international criteria [19]. The control subjects consisted of 23 non-smokers (18 men and 5 women; mean age: 42.8 ± 7 years; range: 38-52 years) undergoing routine investigations for suspected bronchial carcinoma and whose chest X-ray (CXR), bronchial examination, and pulmonary function were normal. None of them had evidence of acute infection or chronic disease (e.g., other autoimmune or atopic disorders). Whole blood (10 BD patients) and BAL (BD-27 patients) were obtained after informed consent. Blood samples (5 ml) were immediately transferred into PAXgene Blood RNA Tubes (Qiagen) for isolation and purification of intracellular RNA. Blood from ten active BD patients (8 patients with asymptomatic functional abnormalities and 2 patients with pulmonary artery aneurysm of varying signs) were studied for NOD1 and NOD2 mRNA expression before and after treatment. Remission in these patients was started as they lost all their clinical symptoms (eye lesions, oral ulcer, genital ulcer and arthritis). Patients with asymptomatic functional abnormalities lost these symptoms but not the two patients who have pulmonary artery aneurysm. They were treated with steroids and colchicine. Our hospital ethic committee approved the design of the study.

### Bronchoalveolar lavage (BAL)

BAL was obtained as we previously reported [17,18]. Briefly, bronchoscopy was performed according to standard guidelines [18]. Thirty minutes prior to the procedure patients received 0.5 mg of atropine and 12.5 mg codeine intramuscularly. Local anaesthesia of the oropharynx was achieved by xylocaine instillation until gag reflexes subsided. Bronchoscopy was performed using a Pentax bronchoscope through which 150 ml of normal prewarmed saline in aliquots of 20 ml were instilled into a subsegment of the right middle lobe. BAL fluid was then immediately aspirated by gentle hand suction into plastic tubes and kept at 4°C on ice.

BAL fluid was concentrated 10 fold before analysis whilst a great part of the cell pellets were immediately fixed in RNA stabilisation buffer. The total count of nucleated cells was performed as we have recently reported [20]. Differential cell counts were performed on cytospin slides. Following Pappenheim staining, 300 cells per slide were counted. Lymphocyte counts in BAL (Table 1) were confirmed by flow cytometry (10^4 ^gated events) after staining 5 × 10^5 ^cells with anti CD4-FITC, anti CD8-PE and anti CD3-PercP antibodies (BD Biosciences, UK).

**Table 1:** Patients and controls characterization

BAL analysis	Behçet's Disease (n = 27)	Sarcoidosis (n = 8)	Healthy Controls
% Recovery	67 (59-77)	68 (65-74)	69 (63-76)

% Viability	95 (93-97)	94 (92-97)	92 (90-96)

Cell concentration (*10^6^/L)	182 (150-320) ¤	175 (110-325)¤	120 (109-270)

Total cell number (*10^6^/L)	23 (17-32)¤	26 (18-40)¤	15 (9-27)

BAL differential cell counts			

% Macrophages	74 (58-77)	77 (62-84)	65 (55-67)

% Lymphocytes	19 (14-28) ^¤^	22 (17-30)¤	9 (7.5-12)

% Neutrophils	0.7 (0.5-1.2)	1.4 (0.9-1.8)¤	0.4 (0.2-0.6)

% Eosinophils	0 (0-0.5)	0 (0-0.7)	0 (0-0.2)

CD4: CD8 ratio	4.2 ± 0.7 (3.8-5.4)¤	6.5 (3.6-9.8)¤	2.3 ± 0.5 (1.4-2.9)

copy number IFNγ	2786 ± 748 (1097-3600)¤	3247 ± 921 (2568-4500)¤	18 ± 9.5 (12-127)

BAL Th1/Th2 ratio	4.5 ± 1.2 (2.8-5.3) ¤	5.8 ± 4.3 (5.2-12.8) ¤	1.3 ± 0.7 (0.5-1.9)

Values are presented as median ± SD (ranges) or median (range). (¤): Significance Behçet disease versus control subjects, (*P = 0.001*). BAL samples from 27 BD patients with pulmonary involvement, 8 patients with sarcoidosis and 23 controls were investigated. The pulmonary arteries are the second most common site of arterial involvement, preceded by the aorta. Aneurysms are more common than thrombosis.

Anti-human macrophage, CD68 monoclonal antibody (PG-M1) was used to characterize the macrophages phenotype recovered with BAL. The staining was performed using a three-step, indirect immunoalkaline phosphatase (ALP) method. Frozen slides were slowly warmed to room temperature before processing. Slides were fixed in -20°C acetone for 10 min and rehydrated for 5 min in Tris-buffered saline (TBS), pH 7.6, containing 1% bovine serum albumin (BSA). The slides were then incubated with 80 μl of the appropriate dilutions of: CD68 monoclonal antibody, ALP-conjugated rabbit anti-mouse antibody (Dako, Glostrup, Denmark) and ALP-conjugated swine anti-rabbit antibody (Dako), 30 min each. The incubations took place in humid chambers and the samples were carefully washed with TBS between the steps. The immunological reaction was visualized using freshly prepared ALP substrate (Phosphatase Fast Red Sigma solution, Sigma) containing 1 mmol/L levamisole (Sigma) to inhibit endogenous macrophage ALP activity. The enzyme-substrate reaction was interrupted with tap water and the slides were counterstained with Harris Haematoxylin (Histolab, Gothenburg, Sweden) for 30 s, blued in tap water and air-dried. For mounting, Glycerine Mountant (Merck, Darmstadt, Germany) was used. The slides were viewed with a Nikon light microscope. The macrophages were identified on the basis of morphological features and positive cells were recognised by red staining. An irrelevant monoclonal antibody (anti-human cytokeratin; Dako) negative for macrophages was used to assess background staining.

### Reverse transcription and real-time PCR

RNA was isolated from whole blood and from lavage cell pellets using the Paxgene^® ^and RNeasy^® ^Kit, respectively. Reverse transcription and real-time PCR, to quantify mRNA encoding for NOD2, NOD1, IL-4, IFN-γ and TLRs, were performed on samples, as previously described [20], after quality control of RNA templates [21]. mRNA values were normalised to a validated housekeeping gene, human-acidic-ribosomal-protein (HuPO) [22]. Primer and probe sequences are shown in Table 2.

**Table 2:** Primer and probe sequences used to quantify gene expression by real-time polymerase chain reaction (PCR)

Genes	Δ Probe sequence 5'-(FAM - TAMRA)-3'	Product size
	• L primer-5'-3'	
	■ R primer-5'-3'	
IL-4	Δ AAACCTTCTGCAGGGCTGCGAC	71 bp
	• GCTGCCTCCAAGAACACAAC	
	■ CTGTAGAACTGCCGGAGCAC	

NOD1	Δ CCTGGCTCCGACATCGGTGA	133 bp
	• AAGCGAAGAGCTGACCAAAT	
	■ TCCCAGTTTAAGATGCGTGA	

NOD2	Δ CCGAGGCATCTGCAAGCTCA	82 bp
	• CTGCAAGGCTCTGTATTTGC	
	■ CTCGCAGTGAAGAGCACATT	

HuPO	Δ TGCCAGTGTCTGTCTGCAGATTGG	105 bp
	• GCTTCCTGGAGGGTGTCC	
	■ GGACTCGTTTGTACCCGTTG	

TLR2	Δ GGAGTTCTCCCAGTGTTTGG	105 bp
	• GCATTGTCCAGTGCTTCAAC	

TLR4	Δ AGTGAGGATGATGCCAGGAT	147 bp
	• TTCATGCCAGCTCTTCTGTG	

HuPO: human acidic ribosomal protein; IL: interleukin.

### Quantification of T-bet gene expression by RT-PCR

The expression of mRNA was quantified using the Applied Biosystems 7500 Fast Real-Time PCR System (Applied Biosystems, Foster City, CA, USA) as we have recently reported [20]. Amplification of cDNA was performed with the TaqMan Universal PCR Master Mix (2×), No AmpErase UNG (Applied Biosystems). A reaction volume of 25 μl (1.0 μl cDNA) was amplified for 40 cycles of 10 s at 95°C and 1 min at 60°C. All samples were analysed in duplicate, and transcription expression was calculated as a mean and standard deviation (SD). For quantification of cDNA a five point serially four-fold diluted standard curve was developed from CSF cell cultures stimulated with phytohaemagglutinin (PHA). The mRNA expression of the T cell transcription factors was standardized to 18S (human rRNA) and all results are expressed as a ratio. A coefficient of variance < 15% was accepted as maximum variation among duplicates. The intra-assay variance for 18S was 4.9%, and T-bet 6.2%. Samples revealing an undetectable expression in both duplicates in three subsequent analyses were assigned an expression quantity of zero. Primers and probes for T-bet expression were analysed with TaqMan^® ^Gene Expression assays (Applied Biosystems). The following sequences were used: T-bet (sense: GCGCCAGGAATTTCATTT, and antisense: CATTCTGGTAGG CAGTCACG). HPLC-purified oligonucleotide primers and probes were bought from MedProbe (Oslo, Norway). All in-house designed mRNA amplicons included at least one exon-exon boundary to assure specificity (marked in bold in the sequences above), and reaction concentration was optimized prior to analysis of samples.

### Flow cytometry analysis

BAL cells were analyzed for expression of NOD1 and NOD2 proteins on a Coulter Epics XL flow cytometer (Beckman Coulter, INSERM 354, Paris, France) and gated based on forward and side scatter properties. Data were analyzed with the Expo32 ADC analysis software (Beckman Coulter). To detect the intracellular NOD1 and NOD2, the IntraPrepTM Permeabilization reagent kit was used according to instructions of the manufacturer (Beckman Coulter). Unlabeled NOD2 monoclonal antibody (mAbs) was detected using the Alexa Fliuor 488 mouse IgG1 labelling kit (Momecular Probes, Eugene, OR, USA). The rabbit anti-human NOD1 pAb was identified using a secondary fluorescein isothiocyanate (FITC)-conjugated goat anti-rabbit IgG pAb. Cells were incubated with Abs for 30 min, thereafter washed and resuspended in PBS (phosphate buffered saline).

### Data analysis

#### Statistical analysis

All data are reported as medians (ranges, minimum to maximum). Differences between groups were analyzed by using the Wilcoxon signed-rank test or the Mann- Whitney *U *test. Correlation was assessed using Spearman rank test. A *P *value of less than 0.05 was considered significant.

## Results

1. Broncho-alveolar lavage (BAL) fluid analyses

BAL fluids recovered from BD and sarcoidosis patients were more cellular than healthy controls, containing significantly greater number of lymphocytes (P < 0.05) (Table 1). BD-BAL macrophage showed similar CD68 expression (33.46% ± 5.9%; range: 27-42) when compared to BAL from healthy controls (35.47% ± 6.2%; range: 25 - 40; *P *>*0.05*). In contrast BAL-macrophages from sarcoidosis patients expressed higher CD68 positive cells than in BD (56.43% ± 9.8%; range 40 - 61.5; *P *= *0.0002*). In BAL-BD and sarcoidosis patients the Th1/Th2 (IFN-γ/IL-4) ratio and IFN-γ mRNA levels were significantly elevated compared to healthy controls (Table 1).

2. BAL from BD patients express NOD2 mRNA

**Figure 1:**
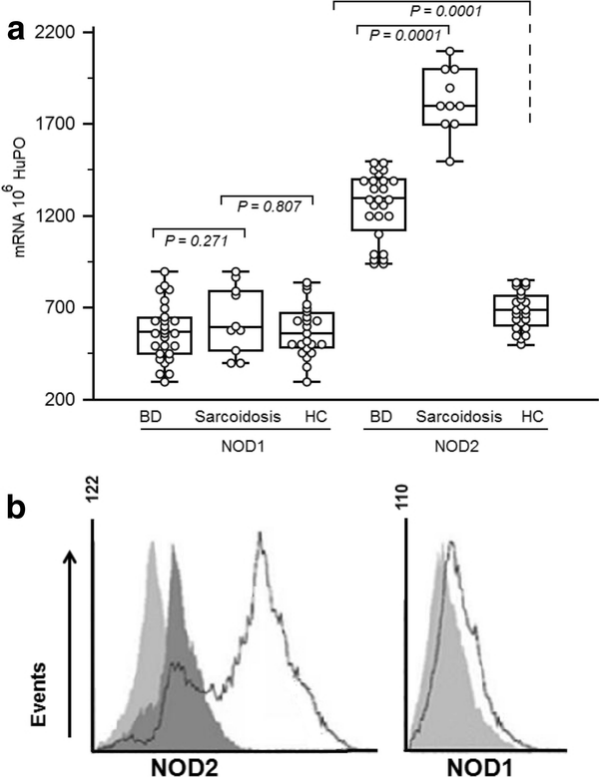
**NOD1 and NOD2 mRNA expression in BAL leucocytes obtained from Behçet's disease (BD) patients**. [**A**]: NOD1 and NOD2 mRNA expression in BAL from BD and sarcoidosis patients compared to healthy controls. NOD1 and NOD2 mRNA expression were measured by real-time RT-PCR. To permit comparison between individuals, absolute copy numbers of NOD1 and NOD2 mRNA were measured during RT-PCR, using cDNA standards, and expressed relative to 10^6 ^mRNA copies of a validated housekeeping gene, HuPO. [**B**]: Detection of NOD1 and NOD2 protein in bronchoalveolar lavage cells from BD patients by flow cytometry. Histograms shown are representative of two independent experiments. Staining against NOD1 and NOD2 (open histogram), appropriate isotype controls (light shaded histogram) or secondary Abs (dark shaded histogram). Data show one representative out of three independent experiments

NOD2 mRNA expression levels are elevated in pulmonary leucocytes from BD and sarcoidosis patients and differ significantly when compared to healthy controls (Figure 1A). NOD2 mRNA was higher in sarcoidosis patients than in BD patients (*P *= *0.0001*). We also compared the expression levels of NOD1 mRNA (also known as CARD4), in pulmonary leucocytes obtained from BD and sarcoidosis patients and healthy controls. NOD1 is an intracellular protein that is closely related to NOD2. No differences were observed in NOD1 expression between BAL-BD patients, BAL-sarcoidosis patients and BAL-healthy controls (Figure 1A). At the protein level, a clear NOD2 up-regulation was observed in BAL of BD patients. No NOD1 staining was seen (Figure 1B).

3. NOD2, and TLR2 and TLR4 mRNA expression correlate in BAL-leucocytes in BD patients.

**Figure 2:**
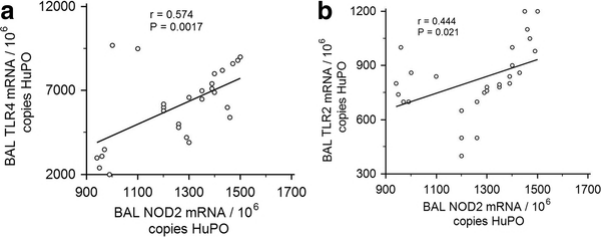
**NOD2 mRNA expression correlates significantly with TLR2 and TLR4 mRNA expression in BAL leucocytes harvested from Behcet's Disease (BD) patients with pulmonary manifestations**. [**A**]: Significant correlation was observed between NOD2 and TLR4 mRNA expression, and [**B**]: NOD2 and TLR2 mRNA expression. To permit correlations, absolute copy numbers of NOD2 and TLR2 and TLR4 mRNA were measured during RT-PCR, using cDNA standards, and expressed relative to 10^6 ^mRNA copies of a validated housekeeping gene HuPO. NOD2 mRNA expression correlates significantly with TLR2 and TLR4 mRNA expression

NOD2 regulates cellular responses to peptidoglycan-mediated activation of TLR2 [23]. TLRs are associated with Behcet's disease pathogenesis [24,25]. In pulmonary leucocytes obtained from BD patients, NOD2 mRNA expression correlates significantly with TLR4 (r = 0.574; P = 0.001) and TLR2 (r = 0.444; P = 0.021) (Figure 2A; 2B). In healthy controls, there was no correlation between NOD2 and TLR2 or TLR4 mRNA expression in BD-pulmonary leucocytes. NOD1 mRNA expression correlates significantly with TLR2 (r = 0.489; P = 0.0095) and TLR4 (r = 0.574; P = 0.0017) mRNA expression in pulmonary leucocytes obtained from BD patients and healthy controls.

4. Quantification of T-bet mRNA expression in BAL cells from BD patients

**Figure 3:**
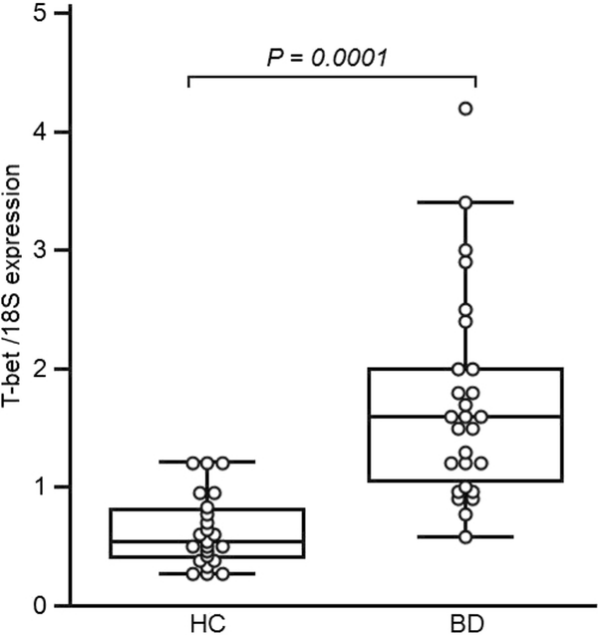
**Transcription factor expression folds change in BAL from patients with Behçet's Disease (BD), and healthy controls**. All values represent quotes between transcription factor and 18S expression. In the figure, the horizontal bars within boxes correspond to the median; box limits correspond to 25th and 75th percentiles and vertical lines indicate the range. The mean values were compared and the *P *values are indicated at the figure. Significant difference was observed in T-bet value between BAL-BD patients and BAL-HC

We evaluated mRNA expression of BAL samples from BD patients and healthy controls (Figure 3). T-bet mRNA was constitutively expressed in all BAL cell samples obtained from BD patients and healthy controls. There was a significant increase of T-bet mRNA expression in BAL from BD patients compared to BAL from healthy control (*P *= *0.001*). We did not observe any difference in the number of T-bet transcripts and the different pulmonary manifestations in BD.

5. Correlation of T-bet mRNA expression with BAL cellular profile in BD

In order to find out whether T-bet expression correlates with the BAL cellular profile, we performed correlation analysis. T-bet mRNA expression correlates with BAL lymphocyte count (r = 0.684; *P *= *0.0001*) in BD. A low and significant correlation was observed between BD-BAL macrophages and T-bet mRNA expression (r = 0.393; *P *= *0.045*).

In patients with BD, a significant correlation was found between NOD2 mRNA expression and BAL-lymphocytes (r = 0.485; *P *= *0.010*). We did not find any correlation between BAL-macrophages and NOD2 mRNA expression (r = 0.217; *P *>*0.05*). A strong correlation was observed between T-bet and NOD2 mRNA levels in BAL from BD patients (r = 0.602; *P *= *0.0009*).

6. NOD2 expression in peripheral blood

**Figure 4:**
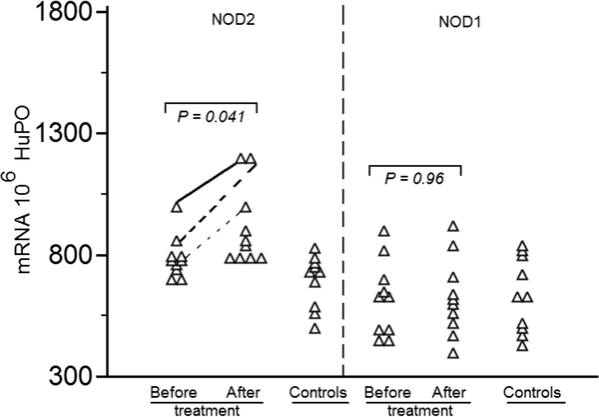
**Comparative analysis of NOD1 and NOD2 mRNA expression in peripheral blood mononuclear cells (PBMC) obtained from 10 active Behcet disease (BD) patients with pulmonary manifestations before and after treatment after their symptoms had resolved**. NOD1 and NOD2 mRNA expression was analysed, by real-time RT-PCR, in peripheral blood cells isolated from BD patients and matched controls. To permit comparison between individuals, absolute copy numbers of NOD1 and NOD2 mRNA were measured during RT-PCR, using cDNA standards, and expressed relative to 10^6 ^mRNA copies of a validated housekeeping gene HuPO. NOD2 in 3/10 patients but not NOD1 mRNA levels increased significantly after treatment. Statistical significance was evaluated by the Wilcoxon test

NOD2 was increased in PBMC from BD patients (791.90 ± 87.49) with low significance when compared to healthy controls (695.0 ± 108.75; *P *= *0.042*). No significant difference was observed in NOD1 mRNA expression between BD patients (624.30 ± 153.18) and healthy controls (636.0 ± 160.2; *P *= *0.86*) (Figure 4).

PBMC of 10 BD patients with pulmonary manifestations were tested for NOD1 and NOD2 mRNA expression before and after treatment (Figure 4). All 10 BD patients were on colchicine (1-2 mg/day). Additionally, 7 patients received cyclosporine (5 patients received 50-100 mg/day and 3 patients received 200 mg/day). NOD1 and NOD2 exhibited similar mRNA expression before and after treatment.

No difference was observed in NOD1 mRNA expression before and after treatment. Significant differences in NOD2 mRNA expression were observed in 3/10 BD patients after treatment. These patients were characterized by receiving high dose of corticosteroids.

## Discussion

Although the aetiology of BD is not yet known, it is thought that genetic predisposition and immune dysregulation are critical factors in the pathogenesis. Most of the studies reported that BD is driven primarily by proinflammatory and Th1 cytokines. NOD2 is a member of the phylogenetically conserved NLR (NACHT-leucine-rich repeat) family, that acts through NF-KB modulation. To further define a role for NOD2 in BD with pulmonary manifestations, we analysed NOD2 transcriptional responses in pulmonary leucocytes and mononuclear cells. As a systemic inflammatory disease, sarcoidosis NOD2 mRNA expression in BAL was compared to BAL from BD patients. NOD2 mRNA was highly expressed in BAL from BD patients but at a lower level than in sarcoidosis patients. The expression of NOD2 protein was demonstrated in BAL cells using flow cytometry. NOD2 mRNA expression correlated with TLR2 and TLR4 mRNA expression in BD patients with pulmonary manifestation. The increased NOD2 mRNA expression could be associated to inflammation in the lung from BD patients.

T-bet is expressed preferentially by Th1 and natural killer (NK) cells, and its expression correlates to some extent with that of IFNγ [26]. Numerically and functionally increased Th1 cells are implicated in the development of a variety of auto-immune/inflammatory diseases including BD. In comparison to control subjects, T-bet mRNA expression was increased in BAL from BD patients, suggesting a role for this transcription factor in the immunopathogenesis of Th1 diseases. There is a common agreement on the predominance of Th1 and Th17 cytokines producing cells at the site of inflammation in BD [20,27,28]. Li et al. reported a significantly increased T-bet mRNA expression in the peripheral circulation of patients with active Behcet's disease [29]. Increased levels of T-bet have been reported also in other Th1 diseases such as Crohn's disease [30], sarcoidosis [31] and celiac disease [32]. Recently Kriegova et al. reported that T-bet was detected in lung macrophages and lymphocytes [31]. NOD2 mRNA expression in BD-BAL fluid was correlated with T-bet expression. In view of the crucial role of T-bet in Th1 differentiation and NOD2 mRNA upregulation, we hypothesized that they were involved in the pathogenesis of BD-lung inflammation. Significant correlations were found between NOD2 mRNA expression, T-bet mRNA expression and BAL-BD-lymphocytes.

Macrophages from BD patients expressed similar CD86-positivity than healthy controls. In contrast sarcoidosis patients showed a higher expression of CD68-positive cells. CD68 is a good marker for alveolar macrophages and it is not modulated in lung diseases as reported by St-Laurent et al. [33]. According to this preliminary data, we could speculate that macrophages from BD patients are not directly implicated in the NOD2 production. However, our conviction was that all inflammatory cells contribute to BAL-immune cell activation: macrophages, lymphocytes and dendritic cells [18,28]. Recently we reported that BAFF levels were significantly increased in BAL fluid from BD patients and that BAFF was produced locally in the lower airway of active BD. In the same way IL-6 and IL-13 were also produced locally, and their concentrations were strongly correlated with BAFF concentrations in BAL fluid from BD patients with lung involvement [18].

Autoinflammatory diseases were characterized by seemingly unprovoked episodes of inflammation, without high levels of autoantibodies or antigen-specific T cells, and derive from genetic variants of the innate immune system. Categories of autoinflammatory diseases were associated with NOD2 gene mutation [34]. BD shares clinical features with autoinflammatory disorders [35]. In these autoinflammatory diseases, IL-1β, IL-6 and TNF-α are key cytokines responsible in autoinflammatory disorders. These cytokines and their genetic polymorphism were highly associated with the inflammation in BD patients [5].

Normal levels of NOD2 mRNA expression were found in peripheral leucocytes from BD patients. We hypothesise that this could have been due to translocation of antigen-specific leucocytes predominantly to the site of disease (lung) with few NOD2 expressing leucocytes in the peripheral compartment. We might expect this to occur in parallel with TNF-α as this cytokine up-regulates NOD2 mRNA expression in various cell lineages including PBMC [36]. In keeping with these data, increased TNF-α mRNA expression in BD patients was reported [37]. Akmen et al. [38] reported that TNF-alpha-1031 C allele is associated with susceptibility to BD. They hypothesize that it has a functional effect and could explain the high levels of TNF-alpha production observed in BD patients. The relationship between NOD2 and soluble TNF-α receptors must be investigated.

Crohn's disease (CD) has been associated with two SNPs and a frameship mutation of the CARD15 gene [12]. Despite pathological similarities between BD and CD, contrasting results were reported in BD. Depending on the studied population, no associations as well as negative associations were reported [39,40]. Kappen et al. [41] observed a significantly lower frequency of two variant alleles (Arg702Trp and Leu1007fs) in the BD group as compared with healthy controls. As far, no genetics basis seems involved in surexpression of NOD2 in BD. The complex role of NOD2 in response to bacterial challenge in different cell types should be addressed in BD and other inflammatory conditions. NOD2 variants react in some cases with a decreased production of inflammatory cytokines to an exogenous stimulus. This is supported by the observation that NOD2 gene-deficient mice show reduced joint inflammation, and are protected against early cartilage damage after IA injection of Streptococcus pyogenes cell wall fragments [42]

Tissue repair and remodelling are crucial to the pathogenesis of lung inflammation as well as to host defence, and based on data reported by Lafferty et al. [43], it appears that TLR-dependent mechanisms mediate the development of both processes. In Behcet's disease NOD2 and TLRs appear to serve as independent, pattern recognition receptor of unknown antigens. Recently as reported by Nara et al. [9] TLR-2-expressing cells as well as TLR-4-expressing cells were accumulated in the intestinal lesions of BD. Durrani et al. [44] investigated TLR expression in BD tissue and found it increased in buccal lesions. Similar results were observed in patients with lichen planus or pyogenic granuloma, suggesting that this was a generalized inflammatory response as opposed to a BD-specific response [44]. The initiating cause of BD is unknown, but an aberrant response to infection has been suggested [45]. Because TLRs are likely to be involved in the pathogenesis BD [9,24,25,44], further investigation of molecular mechanisms, including interactions between TLRs and NOD2, are required, especially those that distinguish BD from other inflammatory diseases.

This study has limitations. First, we have used RT-PCR measurement of T-bet and NOD2 ex vivo to provide information about their possible relationship in BD with pulmonary manifestations, but we did not prove the mechanism of interaction between these related genes by functional studies. Secondly, this study focused on the NOD mRNA expression profiles only in BAL cells and not in lung parenchyma. Third, NOD2 and T-bet must be investigated in purified BAL-cells: macrophages and T lymphocytes. However, important preliminary data were found. Significant correlations between NOD2 and T-bet mRNA expression and T-bet mRNA expression and BAL-lymphocytes count were found.

## Conclusions

In summary, this is the first study suggesting implication of T-bet, TLRs and NOD2 system in BAL-BD patients. It is clear that BD is a complex disorder with possible involvement of multiple genes and pathways along with contribution from environmental factors. Proteins regulating the inflammation process via the NF-KB pathway are probably essential in Behçet's disease pathogenesis.

## Abbreviations

BAL: Bronchoalveolar lavage; CD: Crohn's disease; IA: Intra-articular; MDP: Muramyl dipeptide; MNC: Mononuclear cell; NOD: Nucleotide-binding oligomerization domain; PBMNC: Peripheral blood mononuclear cell; PRR: Pattern recognition receptor; SD: Standard deviation; TLR: Toll-like receptor; TNF: Tumor necrosis factor; T-bet: T-box expressed in T cells.

## Competing interests

The authors declare that they have no competing interests.

## Authors' contributions

AH, KH: participated in the design and coordination of the study and to manuscript writing, performed the experiments and analyzed the data. AB, JA and HA: participated in the design, coordination of the study and analyzed the data. All authors read and approved the final manuscript.
